# Changes in the nanoparticle aggregation rate due to the additional effect of electrostatic and magnetic forces on mass transport coefficients

**DOI:** 10.1186/1556-276X-8-20

**Published:** 2013-01-10

**Authors:** Dana Rosická, Jan Šembera

**Affiliations:** 1 Technical University of Liberec, Institute of Novel Technologies and Applied Informatics, Liberec 460 01, Czech Republic

**Keywords:** Magnetic nanoparticles, Aggregation, Mass transport coefficient, Limit distance

## Abstract

The need may arise to be able to simulate the migration of groundwater nanoparticles through the ground. Transportation velocities of nanoparticles are different from that of water and depend on many processes that occur during migration. Unstable nanoparticles, such as zero-valent iron nanoparticles, are especially slowed down by aggregation between them. The aggregation occurs when attracting forces outweigh repulsive forces between the particles. In the case of iron nanoparticles that are used for remediation, magnetic forces between particles contribute to attractive forces and nanoparticles aggregate rapidly. This paper describes the addition of attractive magnetic forces and repulsive electrostatic forces between particles (by ‘particle’, we mean both single nanoparticles and created aggregates) into a basic model of aggregation which is commonly used. This model is created on the basis of the flow of particles in the proximity of observed particles that gives the rate of aggregation of the observed particle. By using a limit distance that has been described in our previous work, the flow of particles around one particle is observed in larger spacing between the particles. Attractive magnetic forces between particles draw the particles into closer proximity and result in aggregation. This model fits more closely with rapid aggregation which occurs between magnetic nanoparticles.

## Background

There are a lot of types of nanoparticles and colloidal particles in groundwater [1]. Some of them are formed naturally, others are generated synthetically and put into the ground by humans. Not only is the reactivity of particles important, but also their migration properties are examined. For example, natural bentonite colloids are released as a consequence of bentonite disposal of radioactive wastes and could carry adsorbed radionuclides in groundwater through granite [2,3]. Zero-valent iron nanoparticles are produced [4-6] and injected into the ground. Iron nanoparticles are able to migrate in groundwater through contaminated areas and remediate the polluted soils and water [7]. In the first case, the migration possibility is unwelcome. In the second case, the better the migration, the more effective of the remediation. That is why a simulation of the migration of nanoparticles might be desirable. To simulate the migration of nanoparticles, the coefficient of transport retardation of the nanoparticles is needed. The coefficient represents the possible reduction in the rate of nanoparticle migration compared with nanoparticles with similar properties. The number of nanoparticles with similar properties changes over time due to aggregation and it influences the results of the migration experiments. A dynamic model of aggregation has to be included in the simulation programme of nanoparticle transport in flowing water. That is why mass transport coefficients are needed. The coefficients represent the frequency of nanoparticle collisions [8,9].

A commonly used model for mass transport coefficients [10,11] in describing aggregation is based on the collisions among nanoparticles caused by heat fluctuation, the velocity gradient of the water in which the nanoparticles are suspended and the different velocities of sedimentation of nanoparticles of varying size. This model does not include the decrease in the rate of aggregation due to repulsive electrostatic forces which occurs due to the electric double layer which builds up on nanoparticle surfaces [12]. Further, in the case of magnetic nanoparticles, the aggregation rate is rapidly increased due to the attractive magnetic forces between nanoparticles [4,13-16]. That is the reason why the model of aggregation has been expanded, enabling a more accurate model of aggregation of iron nanoparticles in water to be achieved. The paper describes the extension of the mass transport coefficients by the attractive magnetic forces and repulsive electrostatic forces between the nanoparticles.

## Methods

### A model of nanoparticle aggregation

Particles aggregate easily in groundwater. They create clumps of particles up to the size of several micrometres [15] that cohere and reduce the ability of particles to migrate through the pores on the ground. The aggregation of the particles is caused by processes that generally occur during particle migration. The reduction in mobility can be formulated by a rate of aggregation given by mass transport coefficients *β* (m^3^s^-1^) [9,10]. The coefficients give a probability *P*_ij_ for the creation of an aggregate from particle *i* and particle *j* with concentrations *n*_i_, *n*_j_ of particles *i*, *j*, respectively (Equation 1). Particle *i* means the aggregate is created from *i* elementary nanoparticles.

(1) $$P_{\text{ij}}=\beta_{\text{ij}}\;n_{\text{i}}\;n_{\text{j}},$$

(2) $$\beta_{\text{ij}}=\beta_{\text{ij}}^{1}+\beta_{\text{ij}}^{2}+\beta_{\text{ij}}^{3}.$$

The coefficient (Equation 2) is given by the sum of mass transport coefficients of Brownian diffusion $\beta_{\text{ij}}^{1}$, velocity gradient $\beta_{\text{ij}}^{2}$ and sedimentation $\beta_{\text{ij}}^{3}$. The concept is adopted from [10].

In the case of small nanoparticles, temperature fluctuation of particles has a significant effect on particle aggregation [17]. Brownian diffusion causes a random movement of the particles and it facilitates aggregation. The mass transport coefficient for the Brownian diffusion [10] is

(3) $$\beta_{\text{ij}}^{1}=\frac{2\;k_{\text{B}}\;T}{3\;\eta }\;\frac{{\left(d_{\text{i}}+d_{\text{j}}\right)}^{2}}{d_{\text{i}}\;d_{\text{j}}},$$

where *k*_B_stands for Boltzmann constant, *T* denotes the absolute temperature, *η* is the viscosity of the medium, and *d*_i_is the diameter of the particle *i*.

Another process causing aggregation is the drifting of nanoparticles in water. Water flowing through a pore of soil has a velocity profile. In the middle of the pore, the velocity of water is highest. Since the particles have different velocities, according to their location in the flow, the particles can move close together and create an aggregate. The mass transport coefficient for the velocity gradients of particles [10] is

(4) $$\beta_{\text{ij}}^{2}=\frac{1}{6}\;G\;{\left(d_{\text{i}}+d_{\text{j}}\right)}^{3},$$

where *G* is the average velocity gradient in a pore.

Particles settle due to gravitational forces. The velocity of the sedimentation varies for different aggregates depending on their size, so particles can move closer together and aggregate. The mass transport coefficient for the sedimentation [10] is

(5) $$\beta_{\text{ij}}^{3}=\frac{\Pi \;g}{72\;\eta }\;(\varrho_{\text{p}}-\varrho )\;{\left(d_{\text{i}}+d_{\text{j}}\right)}^{2}\;|d_{\text{i}}^{2}-d_{\text{j}}^{2}|,$$

where *g* is the acceleration due to gravity, *ϱ*is the density of the medium, and *ϱ*pis the density of the aggregating particles.

### The magnetic properties of nanoparticles

Because of the composition of nanoparticles, every nanoparticle has a non-zero vector of magnetization. According to [15], TODA iron nanoparticles produced by the Japanese company Toda Kogyo Corp. (Hiroshima, Japan) [5], with diameter of 40 nm have saturation magnetization 570 kA/m. This is the value for a substance composed of nanoparticles containing 14.3% of Fe^0^ and 85.7% of Fe_3_O_4_. We use these data for our model. Therefore, we assume the same size magnetization vector for all nanoparticles.

Our model of a magnetic field around an iron nanoparticle is based on the model of the magnetic field around a magnet described in [18]. The electromagnetic potential in the point **r** near a permanent magnet of volume *V* is equal to

(6) $$\phi (r)=\underset{V}{\int }\frac{MR}{R^{3}}\text{d}V,$$

where **M** is the magnetization vector at the point d*V*, the vector **R** is the difference between source of the magnetic field d*V* and the point **r**, *R* is the length of **R**.

The intensity of the magnetic field **H** can be subsequently computed as

(7) H(r)=−grad(ϕ(r)).

Finally, the magnetic force between the source of the intensity of magnetic field **H** and a permanent magnet of volume $\tilde{V}$ with a magnetization vector **M**_0_ at the point **r** is equal to

(8) $$F(r)=-\underset{\tilde{V}}{\int }(M_{0}\cdot \text{grad})H(r)\text{d}\mathrm{V.}$$

In our previous work [19], the scalar potential of the magnetic field around one homogeneous spherical iron nanoparticle with radius *a* located at the point (0,0,0) was derived as follows:

(9) $$\phi (r)=M\;\overset{2\Pi }{\underset{0}{\int }}\overset{\Pi }{\underset{0}{\int }}\overset{a}{\underset{0}{\int }}\frac{\left(x_{3}-r^{\prime }\cos(\theta ))r^{\prime 2}\sin(\theta \right)}{\sqrt[{3}]{{\left(x_{1}^{2}+x_{2}^{2}+x_{3}^{2}-r^{\prime 2}\right)}^{2}}}\text{d}r^{\prime }\text{d}\mathrm{\theta d\varphi},$$

where *a* is the radius of the nanoparticle, and (*x*_1_,*x*_2_,*x*_3_) are the coordinates of the point **r**. Here, the direction of the magnetization vector **M** is set towards *x*_3_, and *M* is the magnitude of the vector **M**.

From Equations 7 and 8, the analytical computation of the magnetic force between two iron nanoparticles can be obtained. Since nanoparticles aggregate, the magnetic force between aggregates must be derived. One aggregate can be composed of millions of nanoparticles. It would be time-consuming and very difficult to analytically compute all these forces. As a consequence, the forces are computed numerically, either as a sum of the magnetic forces between every nanoparticle in one aggregate with every nanoparticle in the second aggregate

(10) $$F=\overset{n_{2}}{\underset{j=1}{\sum }}\tilde{V}(M_{\text{2j}}\cdot \text{grad})\text{grad}\tilde{\phi }(r_{\text{2j}}),$$

or as one magnetic force between two averaged aggregates [20].

(11) $$F≐V_{2}(M_{\text{2A}}\cdot \text{grad})\text{grad}\phi (R,M_{\text{1A}},\sqrt[{3}]{n_{1}}a).$$

where $\tilde{V}=\frac{4}{3}\Pi a^{3}$ is the volume of a nanoparticle, **r**_2j_ is the location of the centre of the *j*-th nanoparticle in the second aggregate, **M**_2j_ is the magnetization vector of the *j*-th nanoparticle in the second aggregate, **M**_1A_ and **M**_2A_ are the averaged magnetization vectors (Equation 12) of the first and the second aggregate respectively, and $V_{2}=\sqrt[{3}]{n_{2}}a$ is the volume of the second aggregate.

The averaged aggregate is a big homogeneous particle with its direction of magnetization vectors **M**_A_ which is computed as a vector sum of the magnetization vectors of all nanoparticles in the aggregate *M*_A_ and computed as an average of the sizes of all nanoparticles divided by the number of nanoparticles in the aggregate *n*.

(12) $$M_{\text{A}}=\frac{\overset{n}{\underset{i=1}{\sum }}M_{\text{i}}}{n}.$$

#### The structure of aggregates

When particles aggregate due to magnetic forces, the rate of aggregation depends on the magnetization vectors of the aggregating particles and on the distance between the particles. The rate of aggregation changes with the changing number of nanoparticles within the aggregates, that is, the changing scale of the structure by order. The model which has been chosen for the structure of an aggregate is a sphere with randomly located nanoparticles within the aggregate, either with random directions of magnetization vectors for every nanoparticle; or with the same direction of magnetization vectors for all nanoparticles in the aggregate. Aggregate structures were assessed in previous work [21]. A more accurate assessment of the most probable structure of an aggregate was performed for this paper in section ‘The structure of an aggregate based on interaction energy’.

### The electrostatic properties of nanoparticles

In an electrolyte, a surface charge builds up on the nanoparticle surface. The surface charge depends on its zeta potential (see e.g. [22]) which is measurable. The zeta potential strongly depends on the pH of the water. The results of this dependence were measured using the Malvern ZetaSizer (Malvern Instruments Inc, Malvern, Worcestershire, UK) as published in [19]. From the zeta potential, the surface potential can be computed, based on the electrical double layer [23,24]

(13) $$\sigma =-\sqrt{8\varepsilon_{0}\varepsilon_{\text{r}}c\;R_{\text{g}}\;T}\text{sinh}\frac{F\;\mathrm{Z\zeta}}{2\text{RT}}$$

where *σ*is the surface charge density of the particle, *c* is the molar electrolyte concentration, *R*_g_ is the molar gas constant, *F* is Faraday’s constant, *Z* is the charge number and *ζ* is the electrostatic potential. The electrostatic force between two particles is equal to

(14) $$F_{c}=\frac{1}{4\varepsilon_{0}\varepsilon_{\text{r}}}\frac{\Pi d_{\text{i}}^{2}d_{\text{j}}^{2}\sigma_{\text{i}}\sigma_{\text{j}}}{D^{2}},$$

where *D* is the distance between the particles *i* and *j*. The electrostatic forces repel nanoparticles with the same polarity and cause a reduction in the rate of aggregation. Inclusion of the dependence is done in section ‘The inclusion of the limit distance into mass transport coefficients’.

### The limit distance

**Figure 1:**
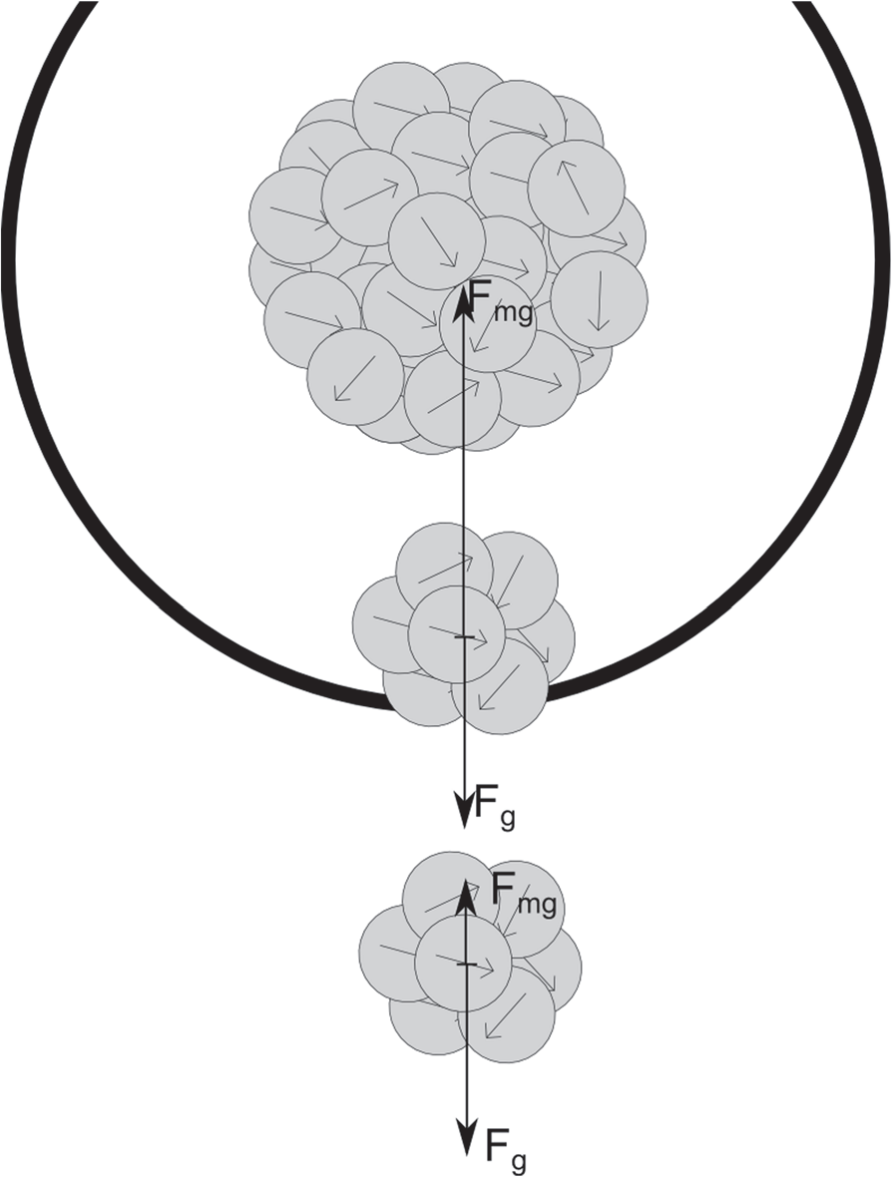
**Sketch of the limit distance.** A comparison of the forces acting on aggregates depicted by a two-dimensional figure. Inside the circle with diameter equal to the limit distance, the magnetic forces outweigh the gravitational force and aggregation occurs. Outside this, the aggregates settle.

The effect of magnetic forces on the rate of aggregation was assessed by one parameter - the limit distance *L*_D_. This dimension expresses the range of magnetic forces between particles. The definition of this parameter is as follows: this is the distance from centre of an aggregate up to which attractive magnetic forces cause the aggregation between the aggregate and a particle placed in this range. Hence, in a range larger than the limit distance, other forces outweigh the magnetic forces (Figure 1). The limit distance *L*_D_ can be defined as the distance of the point in which gravitation *F*_g_ and magnetic forces *F*_mg_ effecting on the aggregate are equal

(15) $$F_{\text{g}}=F_{\text{mg}}(L_{\text{D}}).$$

The limit distance takes the form

(16) $$L_{\text{D,0}}=\;\sqrt[{4}]{\frac{F_{\text{mg}}(R_{0})}{F_{\text{g}}}}R_{0}.$$

The magnetic force between two single domain magnetic nanoparticles falls by the power of 4. In the case of aggregates, the fall depends on the structure of the aggregates and iteration of limit distance computation is needed [20].

(17) $$L_{\text{D,1}}=\sqrt[{4}]{\frac{F_{\text{mg}}(L_{\text{D,0}})}{F_{\text{g}}}}L_{\text{D,0}}.$$

When including electrostatic forces, we define the limit distance as the distance where the repulsive magnetic forces is equal to the sum of attractive forces *F*_mg_ and *F*_C_. As the effect of electrostatic forces falls by the power of 2, electrostatic forces can be included into the equilibrium of force in the following way [20]

(18) $$L_{\text{D,0}}=\sqrt{\frac{\sqrt{F_{\text{C}}^{2}(R_{0})+4F_{\text{g}}F_{\text{mg}}(R_{0})}-F_{\text{C}}(R_{0})}{2F_{\text{g}}}}R_{0},$$

(19) $$L_{\text{D,1}}=\sqrt{\frac{\sqrt{F_{\text{C}}^{2}(L_{\text{D,0}})+4F_{\text{g}}F_{\text{mg}}(L_{\text{D,0}})}-F_{\text{C}}(L_{\text{D,0}})}{2F_{\text{g}}}}L_{\text{D,0}}.$$

The values of magnetization vector and surface charge were selected as follows: **M**=570 kA/m; *σ*=2.5×10^−5^ C/m^2^. We used these selected values for all the computations of the interaction energies and mass transport coefficients.

### Simulation software

All the computations of magnetic forces, limit distance, electrostatic forces and mass transport coefficients were performed using Matlab R2009a software (MathWorks Inc, Natick, MA, USA). The computation was carried out for different sizes of aggregates *i* and *j*, mostly varying in the order of the number of nanoparticles that the aggregates were composed of. The magnetic forces between two aggregates were computed either by summation of the magnetic force between every nanoparticle in the first aggregate and every nanoparticle in the second aggregate (when the ratio *L*_D_/*R*_0_ expresses distance between the aggregates was lower than 15 [20]), or by the averaging of the first and second aggregates. Values for the magnetization vector and surface charge were selected in the following way: **M**=570 kA/m; *σ*=2.5×10^−5^ C/m^2^. For the velocity gradient, we chose the dimensionless value 50. We used these selected values for all the computations of the interaction energies and mass transport coefficients.

## Results and discussion

### The structure of an aggregate based on interaction energy

To assess the most probable structures of aggregates, one can compute an interaction energy *E* between the nanoparticles which make up the aggregate, according to [25]

(20) E=−m·B.

This is the potential energy of the magnetic moment **m** in the externally produced magnetic field **B**. Again, we assume the same magnetization vectors for all nanoparticles in the aggregates with value 570 kA/m [15]. Positive interaction energy means repulsion of the magnetic moment from the magnetic field of another magnetic moment; negative interaction energy means attraction of the dipoles. By summation of the interaction energies between every two nanoparticles in an aggregate, one can deduct the probability of stability of the different structures of the aggregates (the higher the negative interaction energy, the higher the probability of the structure of the aggregate).

**Figure 2:**
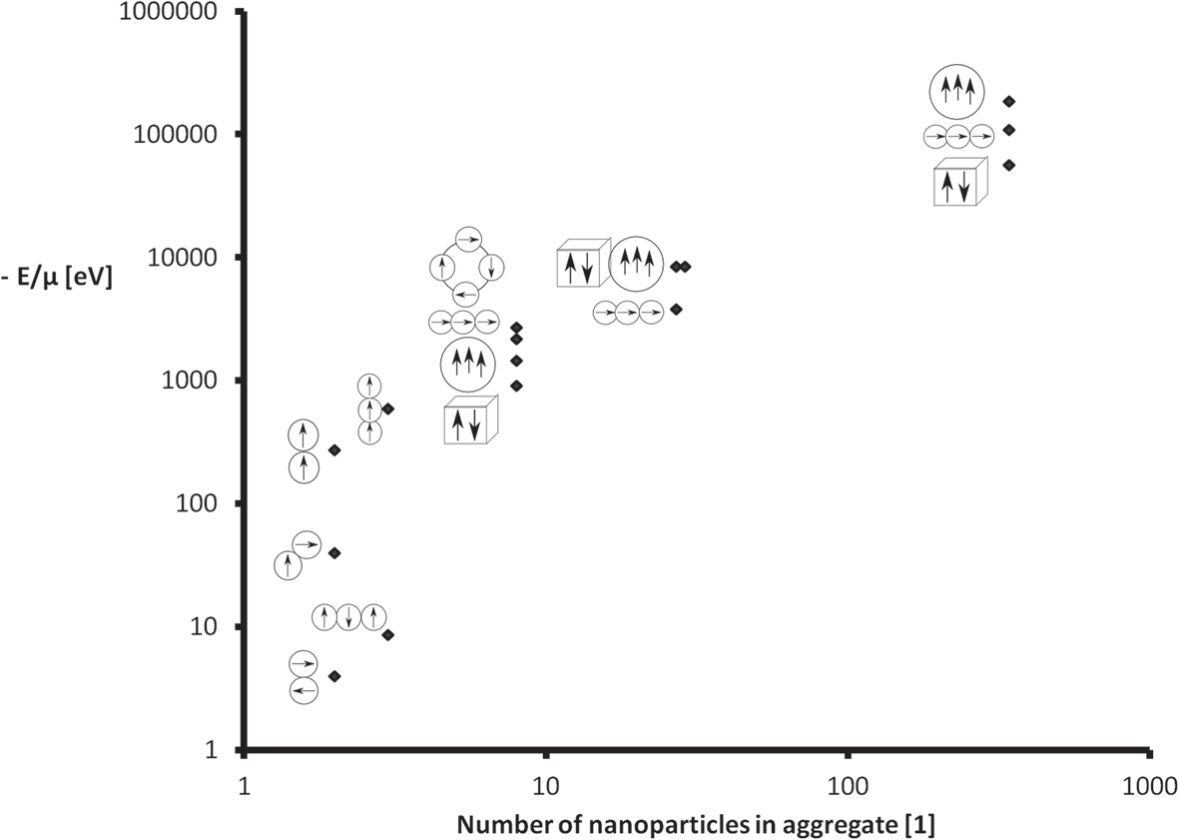
**The interaction energies of different structures of aggregates.** A comparison of the interaction energies of different structures of aggregates expressing the rate of probability of the structures (the larger the negative energy, the bigger the probability of structure).

**Figure 3:**
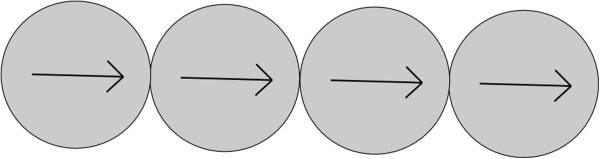
**Diagram of a chain structure.** A diagram of the chain structure of nanoparticles within an aggregate with schematic directions of the magnetization vectors of the nanoparticles.

**Figure 4:**
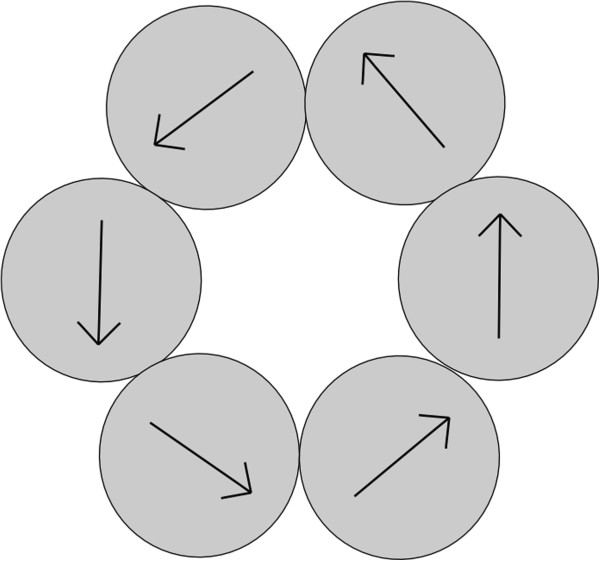
**Diagram of a circular structure.** A diagram of a circular structure of nanoparticles within an aggregate with schematic directions of the magnetization vectors of the nanoparticles.

**Figure 5:**
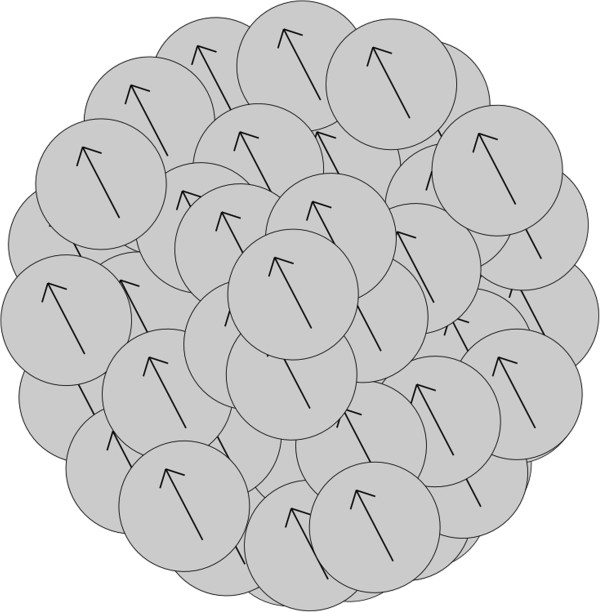
**Diagram of spherical structure.** A diagram of a spherical structure of nanoparticles within an aggregate with schematic directions of the magnetization vectors of the nanoparticles.

**Figure 6:**
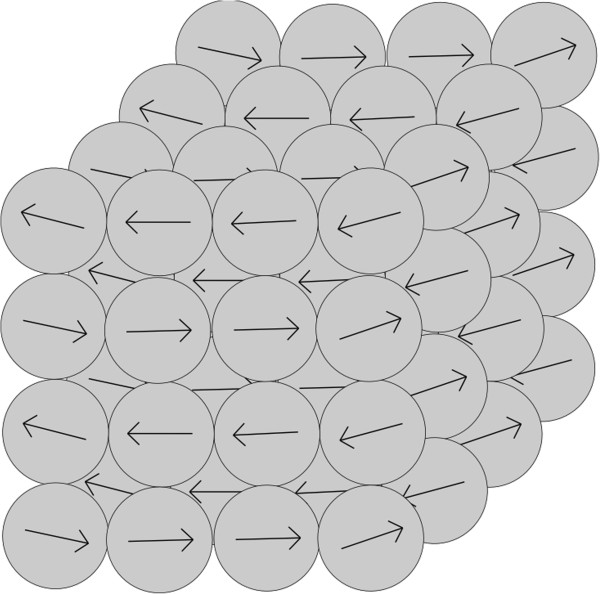
**Diagram of a cubic structure.** A diagram of a cubic structure of nanoparticles within an aggregate with schematic directions of the magnetization vectors of the nanoparticles.

The results of interaction energies are shown in Figure 2. The computed interaction energies are displayed for different structures of aggregates (according to the schemes: Figures 3, 4, 5, 6). The Figure 2 is shown using a logarithmic scale. The exact values of interaction energies for different structures of aggregate (Figures 3, 4, 5, 6) and the different numbers of nanoparticles making up the aggregates are in Table 1. Not the absolute values but the comparison between the values of the different structures is relevant. According to Figure 2, the most probable structure of aggregates for the small aggregates are chains and for the bigger aggregates, spherical clusters with the same direction of magnetization vectors of the nanoparticles which make up the aggregate.

**Table 1:** Interaction energies of different structures of aggregates

**Number of nanoparticles [1]**	**Structure**	**Energy/*μ* (eV)**
2	Chain	273
3	Chain	588
8	Cube	903
8	Sphere	1,449
8	Circle	2,184
8	Chain	2,688
27	Chain	3,780
27	Sphere	8,400
29	Cube	8,400
343	Cube	56,700
343	Chain	109,200
343	Sphere	184,800

Computed interaction energies divided by the permittivity constant for different structures of aggregates (according to the diagrams in Figures 3, 4,5,6) and for different numbers of nanoparticles within the aggregates.

In their research, Phenrat et al. [15], aggregates of nanoscale zero-valent iron particles were measured using dynamic light scattering, optical microscopy and sedimentation measurements. According to their results, firstly, the nanoparticles created clusters and subsequently, these aggregates assemble themselves into fractal, chain-like clusters. We presume that it was because of the high concentration of nanoparticles that they used, and the very fast aggregation, first into chains and then into clusters, which lead to the measurement of only larger clusters in [15]. Our presumption that with larger numbers of nanoparticles, spherical cluster is created which leads to the supposition that at very high concentrations of particles, spherically structured aggregates only attach to each other, without changing their structure. This corresponds to the observations of Phenrat et al. [15]: in high concentrations, first nanoparticles aggregate into clusters, then the created clusters aggregate into pairs or triplets, and finally into chain-like fractal aggregates.

### The inclusion of the limit distance into mass transport coefficients

The basic model of aggregation as given in the section, ‘A model of nanoparticle aggregation’, indicates the rate of aggregation caused by the collision of particles (in proximity, attractive forces outweigh the repulsive ones). We established a limit distance in which attractive forces outweigh the repulsive ones. The magnetic forces attract particles closer to each other and then they aggregate due to attractive van der Waals forces.

Mass transport coefficients (in Equations 3, 4, and 5) were derived on the basis of the flux of nanoparticles through an observed volume or circular area around a particle. The area had a radius equal to sum of the radii of both particles. That means that the particles collide and aggregate. According to our supposition, the particles do not have to be in proximity to aggregate when attractive magnetic forces are acting between them. Therefore, the mass transport coefficients are computed as flux through the spherical or circular area around a particle with a diameter equal to the limit distance:

(21) $$\beta_{\text{ij}}^{1,\text{mg}}=\frac{4\;k_{\text{B}}\;T}{3\;\eta }\;\left(\frac{1}{d_{\text{i}}}+\frac{1}{d_{\text{j}}}\right)\;L_{\text{D,1}},$$

(22) $$\beta_{\text{ij}}^{2,\text{mg}}=\frac{4}{3}\;G\;L_{D,1}^{3},$$

(23) $$\beta_{\text{ij}}^{3,\text{mg}}=\frac{\Pi \;g}{18\;\eta }\;(\varrho_{p}-\varrho )\;|d_{i}^{2}-d_{j}^{2}|\;L_{D,1}^{2},$$

where $\beta_{\text{ij}}^{1,\text{mg}}$, $\beta_{\text{ij}}^{2,\text{mg}}$, and $\beta_{\text{ij}}^{3,\text{mg}}$, stand for the mass transport coefficient of Brownian motion, the velocity gradient, and sedimentation respectively, with the inclusion of magnetic forces between particles. The results of this change in mass transport coefficients are discussed in the next section - ‘A comparison of the rate of aggregation with and without the effect of electrostatic and magnetic forces’.

#### A comparison of the rate of aggregation with and without the effect of electrostatic and magnetic forces

**Figure 7:**
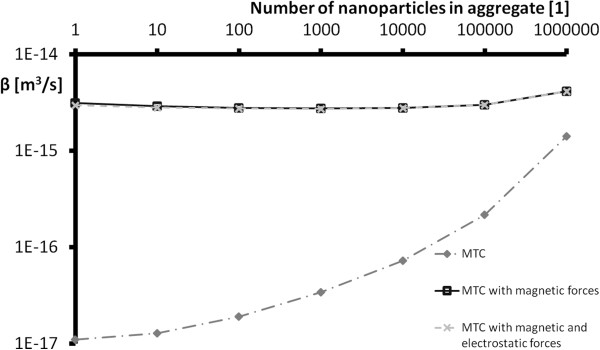
**Mass transport coefficients (MTC) comparison.** A comparison of mass transport coefficients computed by the primary model, mass transport coefficients computed in distance *L*_D_ including magnetic forces, and mass transport coefficients computed in distance *L*_D_ including both magnetic forces and electrostatic forces. The MTC represents the sum of MTCs for Brownian motion, velocity gradient, and sedimentation.

The comparison was carried out using an extreme case with a spherical aggregate structure with the same direction of magnetization vectors of all nanoparticles within the aggregates. The aggregation is highest in this case because attractive magnetic forces attract the aggregates and the rate of aggregation is significantly higher (Figure 7). Table 2 contains a comparison of mass transport coefficients computed by primary model, mass transport coefficients computed in distance *L*_D_including magnetic forces and mass transport coefficients computed in distance *L*_D_including both magnetic and electrostatic forces. The computation of *L*_D_was performed by averaging the magnetic forces for particles with ratio *L*_D_/*R*_0_ higher than 15; otherwise, the computation of magnetic forces was done accurately by summation (for more information see [20]). The values in Table 2 are computed with values **M**=570 kA/m; *σ*=2.5·10^−5^ C/m^2^; *G*=50. According to the results in Table 2 for the chosen values of variables, the attractive magnetic forces between iron nanoparticles have a large effect on the rate of aggregation. The mass transport coefficients are much higher and the aggregation probability increases, which corresponds to our expectations.

**Table 2:** Comparison of mass transport coefficients

***i* [1]**	***j* [1]**	***β*(m^3^*s***^**−1**^**)**	***β***_**mg**_**(m**^**3**^***s***^**−1**^**)**	$\beta_{\text{mg}}^{\text{el}}({\text{m}}^{3}s^{-1})$	$\frac{{L_{\text{D}}}_{\text{mg}}^{\text{el}}}{R_{0}}[1]$
1	1	1.1×10^−17^	3.1×10^−15^	2.9×10^−15^	78.9
1	10	1.3×10^−17^	2.9×10^−15^	2.8×10^−15^	50.6
1	100	1.9×10^−17^	2.8×10^−15^	2.7×10^−15^	28.4
1	1,000	3.4×10^−17^	2.7×10^−15^	2.7×10^−15^	14.6
1	10,000	7.3×10^−17^	2.8×10^−15^	2.8×10^−15^	7.1
1	100,000	2.2×10^−16^	3.1×10^−15^	3.0×10^−15^	3.4
1	1,000,000	1.4×10^−15^	4.2×10^−15^	4.2×10^−15^	1.6
10	10	1.1×10^−17^	1.4×10^−14^	1.3×10^−14^	65.6
10	100	1.3×10^−17^	1.3×10^−14^	1.3×10^−14^	42.0
10	1,000	2.0×10^−17^	1.3×10^−14^	1.3×10^−14^	23.5
10	10,000	4.2×10^−17^	1.3×10^−14^	1.3×10^−14^	12.1
10	100,000	1.6×10^−16^	6.9×10^−14^	6.8×10^−14^	10.2
10	1,000,000	1.3×10^−15^	2.5×10^−14^	2.5×10^−14^	3.2
100	100	1.2×10^−17^	7.1×10^−14^	6.9×10^−14^	54.4
100	1,000	1.5×10^−17^	7.1×10^−14^	7.0×10^−14^	34.7
100	10,000	3.0×10^−17^	7.2×10^−14^	7.1×10^−14^	19.4
100	100,000	1.4×10^−16^	7.0×10^−13^	7.0×10^−13^	21.1
100	1,000,000	1.3×10^−15^	1.9×10^−13^	1.9×10^−13^	6.4
1,000	1,000	1.5×10^−17^	4.0×10^−13^	3.9×10^−13^	45.1
1,000	10,000	3.2×10^−17^	4.0×10^−13^	4.0×10^−13^	28.7
1,000	100,000	1.5×10^−16^	4.1×10^−13^	4.1×10^−13^	16.1
1,000	1,000,000	1.4×10^−15^	1.3×10^−12^	1.3×10^−12^	11.8
10,000	10,000	5.4×10^−17^	2.2×10^−12^	2.2×10^−12^	37.3
10,000	100,000	2.2×10^−16^	2.3×10^−12^	2.3×10^−12^	23.7
10,000	1,000,000	1.8×10^−15^	2.4×10^−12^	2.4×10^−12^	13.3
100,000	100,000	4.4×10^−16^	1.3×10^−11^	1.3×10^−11^	30.8
100,000	1,000,000	2.7×10^−15^	1.3×10^−11^	1.3×10^−11^	19.6

A comparison of mass transport coefficients computed by the primary model *β*, mass transport coefficients computed in distance *L*_D_ including magnetic forces *β*_mg_, and mass transport coefficients computed in distance *L*_D_ including both magnetic forces and electrostatic forces $\beta_{\text{mg}}^{\text{el}}$. The *β* represents the sum of the mass transport coefficients for Brownian motion, velocity gradient and sedimentation. Computation of *L*_D_ was performed by averaging of the magnetic forces (11) for particles with ratio *L*_D_/*R*_0_ higher than 15; otherwise, the computation of magnetic forces was performed accurately by summation (10) (for more information, see [20]). Results were computed using the following values: **M**=570 kA/m; *σ*=2.5×10^−5^ C/m^2^;and *G*=50.

### Discussion

In future work, the system of grouping of particles according to their size will be derived for the new extended mass transport coefficients including electrostatic and magnetic forces. The groups will represent particles with similar transport properties (small particles are easily transportable, large particles remain in the pores in the ground) and a model of aggregation over time will be developed. The model will be compared with the measuring of aggregation of zero-valent iron nanoparticles in time.

Subsequently, the limit distance should be derived for the equilibrium of all forces acting on particles depending on specific conditions.

## Conclusions

In the case of magnetic nanoparticles with non-zero surface charges migrating through the ground, a basic model of interaction between nanoparticles described by the probability of collision due to Brownian motion, velocity gradient, and sedimentation is insufficient. In our previous work, we derived the level of effect of repulsive electrostatic forces between the nanoparticles, and we assessed the level of effect of the attractive magnetic forces between magnetic nanoparticles. In this paper, we summarised the findings and included it into an analytical model of collisions between magnetic nanoparticles. Due to attractive magnetic forces, the rate of aggregation is significantly higher, whereas the repulsive electrostatic forces are almost negligible. One can suppose that with other realistic selections of values of magnetization vector or surface charge, this trend would not change dramatically. This modified model of aggregation can better explain the rapid aggregation of zero-valent iron nanoparticles that is observed. This can help with the simulation of the migration of undissolved particles in groundwater.

## Competing interests

The authors declare that they have no competing interests.

## Authors’ contributions

DR carried out the study of the assessment of the aggregate structure according to interaction energies of the aggregate and with the inclusion of magnetic and electrostatic forces into the aggregation model. JŠ contributed to the conception of the study and to the interpretation of data, and revised the manuscript. Both authors read and approved the final manuscript.
